# “Snapping” of the extensor carpi ulnaris tendon in asymptomatic population

**DOI:** 10.1186/s12891-021-04271-z

**Published:** 2021-04-26

**Authors:** Firat Erpala, Tahir Ozturk

**Affiliations:** 1 Department of Orthopaedics and Traumatology, Cesme Alpercizgenakat State Hospital, 35930 Izmir, Turkey; 2 Department of Orthopaedics and Traumatology, Gaziosmanpasa University, Tokat, Turkey

**Keywords:** Extensor carpi ulnaris, Tendinopathy, Snapping ECU

## Abstract

**Background:**

Extensor carpi ulnaris tendinopathy (ECU) can be one cause of ulnar side wrist pain and it is more prominent in pronation-supination movements against resistance. In supination, flexion, and ulnar deviation within the ulnar groove, the tendon is tense and becomes predisposed to subluxation or dislocation. Snapping occurs during this dislocation and relocation. As a result of this friction between the tendon sheath and ulnar groove, tendinopathy and pain occur. ECU tendon is an important structure that contributes to the dynamic stability of wrist therefore resulting degeneration contributes disruption of distal radioulnar joint and causes wrist instability.

**Methods:**

Participants without active wrist complaints who presented to the outpatient clinic between 2019 and 2020 were included. Provocation test was performed and participants with snapping were evaluated with ultrasound to determine subluxation or dislocation. Participants asked to indicate approximately how much time they spent daily on the phone, computer and game console. The distribution of data was evaluated with the Kolmogorov-Smirnov test. Quantitative data that were not normally distributed were evaluated using the Mann-Whitney U test, and Student’s t-test was used for normally distributed data. The Chi-square test was used to compare categorical variables. For all tests, *p* < 0.05 was considered statistically significant.

**Results:**

Four hundred and fifteen women and 340 men were included in the study. Fifty of the 755 participants (6.6 %) had snapping. 22 of the 755 participants (2.9 %) had dislocation or subluxation on ultrasound. Three (13.6 %) participants had dislocation and 19 (%86.4) participants had subluxation on ultrasound. All 50 of the participants with snapping had significant repetitive trauma and sports activities. It was determined that 21 of the 22 participants who were found to have subluxation or dislocation by ultrasound had more than two hours of hobby activity and significantly more participants had more than two hours of activity compared to the group without subluxation or dislocation.

**Conclusions:**

This study with a large number of participants will contribute to the literature in terms of evaluating the contribution of technological devices, such as computers, smartphones, and consoles to chronic wrist pain and the prevalence of ECU snapping in the asymptomatic population.

**Trial registration:**

Date of Approval; 19.02.2019, Approval Number; 19-KAEK-045.

## Background

The extensor carpi ulnaris (ECU) muscle-tendon is responsible for ulnar deviation and extension movements of the carpal bones [1]. It contributes to wrist stabilization; isometric contraction of the muscle stabilizes the distal radioulnar joint, especially in pronation, and the ulnocarpal joint in supination [2, 3]. It also contributes to the strong hand-grasping movement [4, 5]. The term “ECU tendinopathy” was used for describing all painful ECU anomalies resulting from a dysfunctional 6th extensor compartment including subluxation and dislocation [6]. ECU tendinopathy can be one cause of ulnar side wrist pain and it is more prominent in pronation-supination movements against resistance [6–9].

The tendon sheath consisting of thin collagen fibers is primarily responsible for stabilizing the ECU during wrist movements [10]. In supination, flexion, and ulnar deviation within the ulnar groove, the tendon is tense and becomes predisposed to subluxation and dislocation [11, 12]. “Snapping” (popping) occurs during this dislocation and relocation. Due to this instability and friction between the tendon sheath and ulnar groove, tendinopathy and pain occur. ECU tendon is an important structure that contributes to the dynamic stability of wrist therefore resulting degeneration contributes disruption of distal radioulnar joint and causes wrist instability [3, 6].

This study aims to determine the prevalence of ECU snapping through physical examination and provocation tests of participants with no complaints in the general population and to examine tendon subluxation or dislocation by ultrasound in participants with snapping.

## Methods

Informed consent was obtained from the participants before the procedures began. The study was conducted in accordance with the Helsinki Declaration, and approval was obtained from the local ethics committee.

The sample size was based from a previous study [13]. The alpha level was determined as 0.05, the power as 90 % and the confidence interval as 95 %. The current study was determined to include at least 20 participants.

**Fig. 1:**
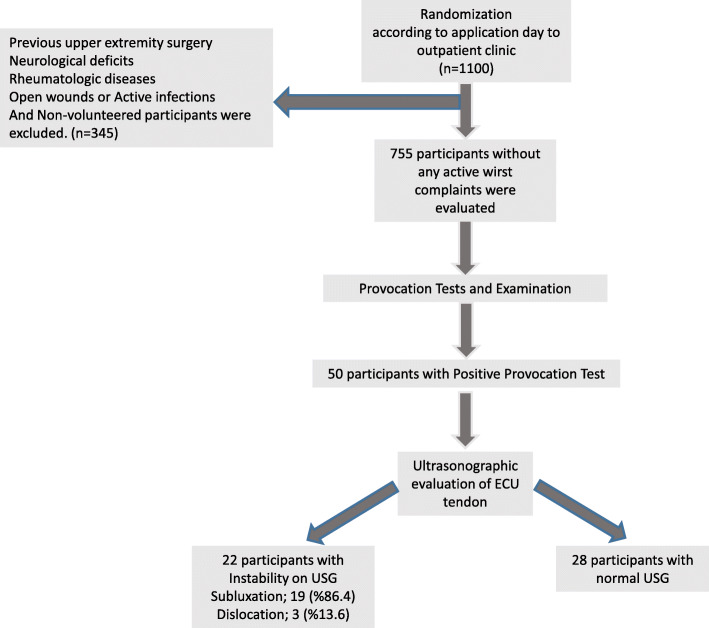
Evaluation of Participants

The study was designed as a prospective randomized study. Participants without active wrist complaints who presented to the orthopedics and traumatology outpatient clinic between 2019 and 2020 were included. Randomization was achieved by including participants who applied on Mondays, Wednesdays, and Fridays in the first and third weeks of the month and participants who applied on Tuesdays and Thursdays in the second and fourth weeks. Participants who applied outside of these dates were not included in the study, even if they met the inclusion criteria. Among the 1100 participants; 755 participants without previous upper extremity surgeries, neurological deficits, rheumatologic diseases, open wounds, or active infections in the wrist area and volunteered to participate in the study were evaluated (Fig. 1).

In the forms prepared for the participants, demographic data, recurrent trauma histories, and sports activity levels were evaluated. They were also asked to indicate approximately how much total time they spent daily on the phone (including social media use, messaging, and game playing), on the computer (mouse use, game playing), and on the game console (joystick use).

For sports activity levels, regular activities for six months or longer were evaluated for sports in which upper extremity activity is intense (including tennis, table tennis, badminton, basketball, volleyball, and bodybuilding).Participants were evaluated independently of their recurrent minor trauma histories, daily housework, and occupational activities other than hobby and sports activities.

**Fig. 2:**
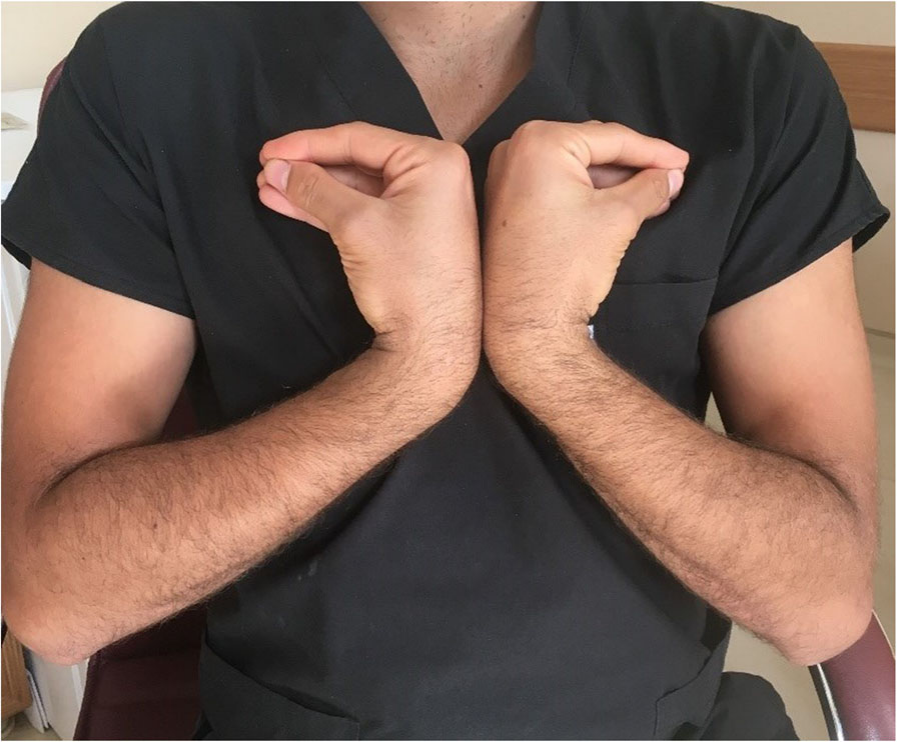
Participant’s “Heart-Shaped” Test

**Fig. 3:**
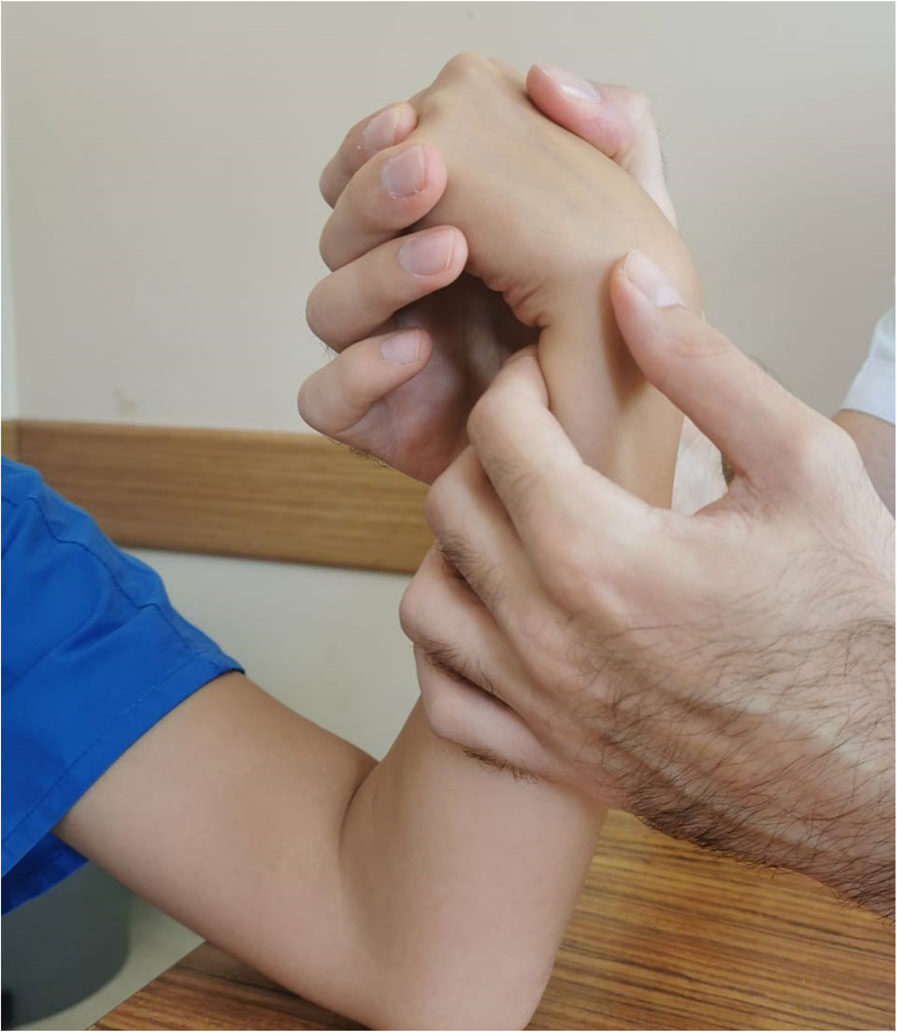
Provocation of ECU snapping by clinician

After obtaining informed consent from the participants, relevant data were entered into the evaluation forms. A bilateral upper extremity provocation test was performed for ECU tendinopathy. The provocation test was first applied to the participants as a “heart-like test” (Fig. 2). They were asked to hold the dorsum of each hand against each other while their wrists were in flexion and their forearms were supine, and then they were asked to press against themselves with the ulnar sides of their hands at chest level. If a snap was not obtained in this way, the clinician performed a provocation test by forcing ulnar deviation against resistance in flexion and supination (Fig. 3). Participants with snapping during the provocation tests were evaluated with ultrasound to determine if there was subluxation or dislocation of the ECU tendon.

Ultrasounds were performed by one radiologist (MMS) with 35 years of professional experience using linear probe (GE Healthcare Systems, Logiq S8). Participants were positioned on examination chair with wrist at flexion and forearm at supination (simulating provocation test). ECU tendon which leaves sulcus approximately 50–99 % was defined as subluxation, tendon which leaves 100 % of the sulcus and stays completely out of the osseous groove, was defined as dislocation [1, 13].

IBM SPSS software (SPSS Inc., Chicago, IL, USA) version 23.0 was used to analyze the data. The distribution of data was evaluated with the Kolmogorov-Smirnov test. Quantitative data that were not normally distributed were evaluated using the Mann-Whitney U test, and Student’s t-test was used for normally distributed data. The Chi-square test was used to compare categorical variables. For all tests, *p* < 0.05 was considered statistically significant.

## Results

Four hundred and fifteen women and 340 men were included in the study. The average age was 38.7 years (18–66 years). Fifty of the 755 participants (6.6 %) had snapping. Ultrasounds of these participants in supination, when the ECU tendon is at its most taut position, were evaluated [10, 11]. Subluxation or dislocation was observed in 22 of the 755 participants (2.9 %) on ultrasound and three (13.6 %) participants had dislocation, 19 (86.4 %) participants had subluxation. The gender, average age, sports activities, and daily computer and phone activity (including social media use, messaging, and game playing) times of the participants with (*n* = 22) and without (*n* = 28) subluxation or dislocation are presented in Table 1.

**Table 1:** Comparison of snapping groups according to presence of instability on ultrasound

Snapping ECU Tendon with Provocation Tests (*n* = 50)			
**Presence of** Subluxation or dislocation **with USG**			
	**Positive (*n* = 22)**	**Negative (*n* = 28)**	***p***
Age	39,09	36,9	0.564
Gender (F/M)	9/13	15/13	0.374
Repetative Trauma (Yes/No)	2/20	3/25	> 0.999
Sports Activity (Yes/No)	4/18	5/23	> 0.999
Hoby Activity (Hours/day)	3.45	2.1	< 0.001

It was observed that all 50 of the participants with snapping had significant repetitive trauma and sports activities. It was determined that 21 of the 22 participants who were found to have subluxation or dislocation by ultrasound had more than two hours of daily hobby activity, and significantly more participants had more than two hours of activity compared to the group without subluxation or dislocation.

## Discussion

Extensor carpi ulnaris tendinopathy is the second most common tendinitis of the wrist. Extensor carpi ulnaris tendon subluxation and dislocation causes instability in the distal radioulnar and ulnocarpal joints [3]. Although ECU instability does not cause symptoms in the early period, over time, symptoms, such as swelling, pain sensitivity, weakness in hand-wrist strength, and a clicking or popping sound, begin to occur on the ulnar side. As the tendon sheath is damaged, it no longer functions properly and the symptomatic subluxation or dislocation becomes chronic.

There is a prevalence study in the literature that was based on a population of professional athletes (such as tennis players, rowers, polo players, and golfers) only [13–15]. In the present study, an asymptomatic population was evaluated, and the plan was to determine the prevalence among the general population.

Since the ECU tendon follows a more linear path in forearm pronation than other positions, it is stably located in the ulnar groove [16]. However, in forearm supination, the ECU tendon tends to be pulled to the radial side under the effect of angular force, and it exits from the ulnar groove [16]. In this study, the position in supination, the tensest position in terms of subluxation and dislocation, was evaluated by examining the ECU tendon position in supination of the participants who had snapping.

In their prospective study, Sato et al. evaluated the ECU synergy test under ultrasound guidance in patients with chronic dorso-ulnar wrist pain. They reported that the test is practical and effective in terms of detecting tendon pathologies [17]. When we evaluated the participants in the present study whose provocation test was positive with ultrasound, we found that 44 % had ECU tendon subluxation or dislocation, even though they were asymptomatic. Again, these participants had significantly higher rates of recurrent trauma and sports activities.

There are studies in the literature on the morphology of the ECU groove that investigated the effects of the depth and morphology of the ulnar sulcus on tendinopathy [18, 19]. Nakashima et al. examined 240 upper extremities and devised four classifications according to sulcus morphology. They stated that 1.3 % were in the classification in which the groove was shallow, and this was observed congenitally regardless of age. In the present study, we evaluated the participants regardless of their ulnar sulcus morphology. Although a flat groove is seen less frequently in the general population, it should be taken into consideration that participants with snapping and instability may have flat or shallow grooves.

According to trends in recent years, we think that the increasing frequency of phone use (messaging with both wrists in ulnar deviation and social media use), the widespread use of game consoles (using joysticks in forearm supination and ulnar deviation), and widespread computer use have increased chronic wrist pain. Burgess et al. stated that approximately 60 % of symptomatic or asymptomatic computer users had distal ECU tendon subluxation or dislocation [20]. In the present study, patients with a minimum of four hours of daily computer use were evaluated. We did not make any limitations when evaluating the daily hobby activity duration. This study found that in participants with snapping, those with an activity duration of more than two hours had statistically significant ECU subluxation or dislocation on ultrasound. Likewise, we found that all but one (95.5 %) of the participants with ECU subluxation or dislocation had a hobby activity habbit of more than two hours per day.

The limitations of this study include the fact that the participants were evaluated independently of ulnar sulcus morphology. Another limitation is that the traumatizing effects of their daily professional activities on the wrist were not evaluated. Although the provocation test was negative it should be kept in mind that mild, asymptomatic subluxation or dislocation, which could theoretically be seen on ultrasound might be evident in the 705 participants.

There is no study on prevalence in the literature, and there is a limited number of published data in the past based on studies involving only professional athletes. We think that this study will contribute to the literature in terms of being a study that represents a technology-dependent asymptomatic population in daily life.

## Conclusions

This is a randomized prospective study with a large number of participants. Based on these features, we think it will contribute to the literature in terms of evaluating the contribution of technological devices, such as computers, smartphones, and game consoles, which are indispensable parts of daily life, to chronic wrist pain and the prevalence of ECU snapping in the asymptomatic population.

## Data Availability

The datasets used and/or analysed during the current study are available from the corresponding author on reasonable request.
